# Notes and News

**Published:** 1904-07-30

**Authors:** 


### Notes and News.

Last week the Prince of Wales, Grand Prior of the Order of St. John of Jerusalem, presented the awards of the Order granted during the past year for life saving, and also service medals for conspicuous services.

Prince and Princess Christian attended the Fancy Fair and Fête at Buckhurst, Wokingham, held in aid of the League of Mercy. The Princess opened the fair on behalf of Princess Victoria of Schleswig-Holstein.

An epidemic of cholera is reported at Bagdad.

The Health Exhibition in connection with the Sanitary Congress at Glasgow was opened last week. The departments include exhibits relating to hospitals and asylums, sick-room appliances, and apparatus in general.

Twenty-four scholarships, 12 fellowships, and 35 grants have been bestowed for the coming year in connection with the Carnegie Trust. The awards will absorb £5,000. The largest number of grants have been made for biological and pathological study.

The Ragged School Union is appealing for funds to secure a fortnight's residence at the seaside for poor London children. The Union has 10 homes, and last year it was able to benefit 6,150 children. The object is one with which all must sympathise, and we hope that much larger contributions will find their way to 32 John Street, Theobald's Road, this year.

In view of the increasing prevalence of cancer, the Committee of the Liverpool Cancer Research Fund are following the example of the Imperial Cancer Research Fund by calling special attention to the importance of early treatment. As they point out, a large number of people put off seeking the advice of their doctor instead of going to him immediately they notice anything wrong. They draw attention to the fact that in men, cancer of the gullet, stomach, and intestines; in women, cancer of the breast and the womb, account for about two-thirds of all cases of cancer.

Mr. Andrew Barlow has presented a further £3,500 to the Royal South Hants and Southampton Infirmary.

The Bishop of London presented prizes to the successful students of St. Bartholomew's Hospital Medical School last week.

A large infant mortality is as conspicuous in the new countries as the old. We learn that in New Zealand 20,000 children between the ages of one and five years have died within a decade.

Mr. T. Atkins Wykes, secretary of the Leicester Infirmary, is resigning his post after 41 years' service. Since he occupied the office the patients increased from 7,000 to 27,000 per annum.

The Norfolk and Norwich Hospital has received a promise of £1,000 from the Duke of Norfolk and of £500 from Dr. Robert Barnes. The Duke's gift will be devoted to an ophthalmic department, and that of Dr. Barnes toward the reconstruction of the museum.

An appeal is being made for new buildings for the Hospital for Invalid Gentlewomen, which is now established at 90 Harley Street. The present hospital is announced to be lacking in modern requirements, and it is stated that a sum of £3,000 to £4,000 will be sufficient to secure an efficient establishment.

A ladies' association has recently been formed in connection with the West London Hospital. Of this Lady Ilchester is a member. She announced that £250 had been secured for the hospital from small fees paid to enter the private gardens at Holland House during the recent Horticultural Show. This experience is suggestive. There are private gardens, picture galleries, and charming houses in the possession of the nobility and of private persons, to inspect which fees would be easily obtainable. Here, then, is a less expensive and more lucrative way of raising money for charity than that afforded by the charity concert, ball, or bazaar.

The important part played by the police in cases of street accident is apparent every day. Seeing how much depends upon the initial handling of an injured person, it is reassuring to know that the police authorities are alive to the responsibilities placed of necessity upon the force. A year ago, the Chief Commissioner of Police intimated that the certificate in first aid of the London School Board, would be recognised as a qualification for promotion. On this announcement being made, 5,000 of the members of the police applied for permission to attend the Board's classes, and a very large number have since been instructed to the benefit of the public. Dr. Collie, who is medical superintendent of the classes, states in an article on the subject, that no attempt is made to manufacture amateur surgeons. The instruction is directed to supply the student with the amount and kind of knowledge only which shall assist him to minimise the dangers of moving the injured, and to render such simple assistance as may be obviously advisable until medical assistance can be procured.

